# Freshwater phytoplankton: biotransformation of inorganic arsenic to methylarsenic and organoarsenic

**DOI:** 10.1038/s41598-019-48477-7

**Published:** 2019-08-19

**Authors:** Hiroshi Hasegawa, Rimana Islam Papry, Eri Ikeda, Yoshiki Omori, Asami S. Mashio, Teruya Maki, M. Azizur Rahman

**Affiliations:** 1 Institute of Science and Engineering, Kanazawa University, Kakuma, Kanazawa 920-1192 Japan; 2 Graduate School of Natural Science and Technology, Kanazawa University, Kakuma, Kanazawa 920-1192 Japan; 3 Faculty of Science and Technology, Federation University, Gippsland, Churchill, VIC Australia; 4 Centre for Environmental Sustainability, School of the Environment, University of Technology Sydney, PO Box 123, Broadway, NSW 2007 Australia

**Keywords:** Environmental impact, Freshwater ecology

## Abstract

The biotransformation and detoxification mechanisms of arsenic (As) species have been active research topics because of their significance to environmental and human health. Biotransformation of As in phytoplankton has been extensively studied. However, how different growth phases of phytoplankton impact As biotransformation in them remains uncertain. This study investigated the biotransformation of As species in freshwater phytoplankton at different growth phases to ascertain at which growth phase different types of biotransformation occur. At the logarithmic growth phase, arsenate (As^V^) (>90%) and arsenite (As^III^) (>80%) predominated in culture media when phytoplankton were exposed to 20 nmol L^−1^ and 1.0 µmol L^−1^ of As^V^, respectively, and methylarsenic (methylAs) species were not detected in them at all. Intracellular As was mainly present in inorganic forms (iAs) at the logarithmic phase, while substantial amounts of organoarsenic (orgAs) species were detected at the stationary phase. At the stationary phase, As^V^ comprised the majority of the total As in culture media, followed by As^III^ and methylAs, although the methylation of As^V^ occurred slowly at the stationary phase. Biotransformation of As^V^ into As^III^ and As methylation inside phytoplankton cells occurred mainly at the logarithmic phase, while the biotransformation of As into complex orgAs compounds occurred at the stationary phase. Phytoplankton rapidly released iAs and methylAs species out of their cells at the logarithmic phase, while orgAs mostly remained inside their cells.

## Introduction

Arsenic (As), an environmental pollutant, is extremely toxic to living organisms at high concentrations. The occurrence of As in aquatic systems is of great concern due to its high bioavailability, bioaccumulation, and trophic transfer from the bases of aquatic food chains through to higher trophic levels^1^. Arsenic in marine biota may not be a significant concern for human health because it is present among them in low concentrations. However, As in freshwater systems is likely to be a significant environmental and human health problem due to the high concentrations that can result from its direct input into these systems from natural and manmade sources^2^. Arsenic exists in different chemical forms in aquatic systems. Two major As species in aquatic systems are arsenate (As^V^), which is the most thermodynamically stable form in oxic waters, and arsenite (As^III^), which is predominant in reduced-oxygen environments^3,4^. Through biotransformation processes, microorganisms like phytoplankton and bacteria can cause significant changes in the biogeochemistry of As in aquatic systems^5–8^. Photosynthetic microorganisms (*e*.*g*., phytoplankton and cyanobacteria) are able to accumulate As^V^ and biotransform it into As^III^ and methylarsenic (methylAs) species, such as monomethylarsonate (MMAA) and dimethylarsinate (DMAA)^9^. Although the toxicity of As^III^ is higher than that of As^V^, As^III^ is predominantly excreted from cells, whereas As^V^ is excreted less. Therefore, a number of researchers have suspected that the reduction of As^V^ to As^III^ represents a detoxification mechanism of phytoplankton^2^.

Arsenic biotransformation by microorganisms plays a significant role in the occurrence, toxicity, and biogeochemistry of this toxic element in the aquatic environment. Several pathways of As biotransformation have been proposed in different microorganisms that are mainly related to oxidation or reduction reactions^10^. The microorganisms conduct these redox reactions either to protect themselves from the toxic effects of this metalloid (as a detoxification mechanism)^11,12^ or to produce energy to promote cellular growth^10,13^. Due to the physicochemical similarities between As^V^ (AsO_4_^3−^) and phosphate (PO_4_^3−^), phytoplankton actively take up As^V^ through the PO_4_^3−^ uptake system, and then biotransform As^V^ inside their cells^14–16^. The high toxicity of As^V^ results because it binds to PO_4_^3−^ receptors that have essential functions inside cells^17^. To reduce its toxicity, phytoplankton biotransform As^V^ inside their cells in a process that involves the two-electron reduction of As^V^ to As^III^, which is mediated by glutathione^18^. The biotransformation of As^V^ into As^III^ and its subsequent methylation to form methylAs species in phytoplankton has been reported in many previous studies^19–23^. Several studies also showed that the rates of As^V^ reduction and methylation in freshwater environments are generally dependent on the occurrence of phytoplankton blooms, which is related to nutrient enrichment and seasonal variables, such as light and temperature^3,6,24^. Sohrin *et al*.^25^ reported an increase in levels of As^III^ in the water during the spring, which was correlated with the growth phases of two distinct phytoplankton blooms. They also observed that PO_4_^3^¯ and As^V^ were also rapidly taken up by phytoplankton cells during these blooms. However, at the stationary bloom phase, when growth is limited by limited nutrient availability, the rates of As uptake and metabolism in phytoplankton were slow, which allowed for the further biotransformation of the pentavalent As^V^ into the trivalent As^III^ and its subsequent methylation to DMAA to occur^17^. Although Sohrin *et al*.^25^ identified a relationship between the growth phases of phytoplankton blooms and the uptake and metabolism of As^V^, little is known about the impacts of different phytoplankton growth phases on As biotransformation inside their cells, as well as the excretion of As metabolites out of their cells. The present study was carried out to address this knowledge gap and reveal the role of phytoplankton in As biogeochemistry in aquatic environments. The diversity in the biotransformation of and behavioral responses to As species by phytoplankton at different growth phases were also reported in this study.

## Results and Discussion

### Growth inhibition effects of arsenic species on freshwater phytoplankton

**Figure 1:**
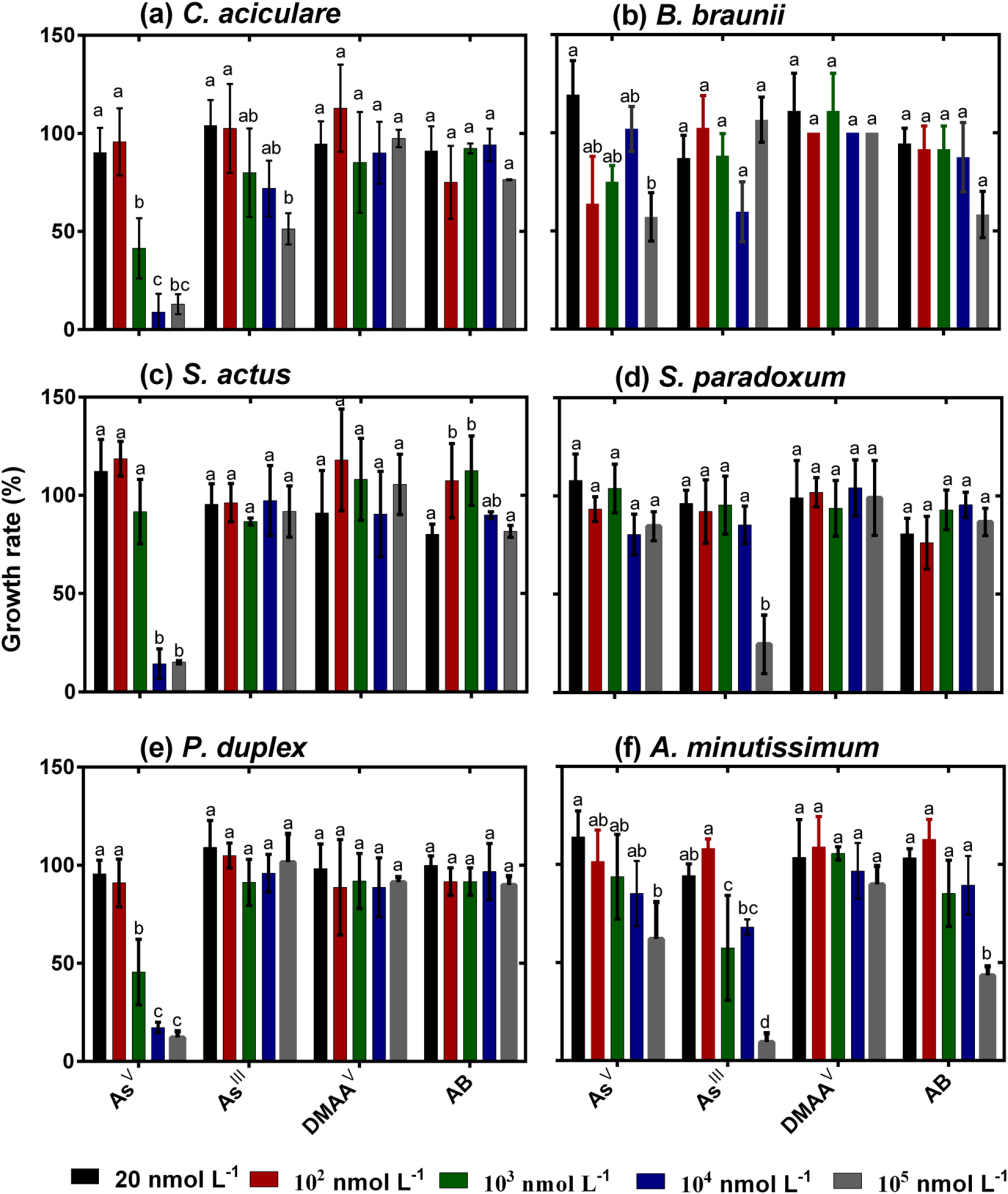
Growth of freshwater phytoplankton under various [As]_0_ treatments. The different lowercase letters indicate significant differences between arsenic treatments (*p* < 0.05). The data presented are mean ± SD growth rates (n = 3).

Inorganic As species had significant growth inhibition effects on freshwater phytoplankton (Fig. 1). In general, the growth of phytoplankton was inhibited substantially by high As^V^ concentrations (≥10^3^ nmol L^−1^), except for *S*. *paradoxum*. The growth rates of *C*. *aciculare*, *S*. *actus*, and *P*. *duplex* were decreased by over 80% in the 10^4^ nmol L^−1^ arsenate treatment (Fig. 1a,c,e). In contrast, the growth inhibition effects of As^V^ on *B*. *braunii*, *S*. *paradoxum*, and *A*. *minutissimum* at a high concentration (10^5^ nmol L^−1^) were less than its effects on *C*. *aciculare*, *S*. *actus*, and *P*. *duplex* (Fig. 1b,d,f). The growth inhibition effect of As^III^ on freshwater phytoplankton was lower than that of As^V^ (Fig. 1). The toxicity of As^III^ reduced the growth rates of *C*. *aciculare*, *S*. *paradoxum*, and *A*. *minutissimum* by between 66 and 80% at an As^III^ concentration of 10^5^ nmol L^−1^ (Fig. 1a,d,f). The freshwater phytoplankton *S*. *actus* and *P*. *duplex* were quite resistant to As^III^ toxicity, even at high concentrations (Fig. 1c,e). These results indicate that As^V^ and As^III^ have different levels of toxicity to different types of freshwater phytoplankton.

Methyl- and organoarsenicals, such as DMAA^V^ and arsenobetaine (AB), respectively, had little growth inhibition effects on the tested freshwater phytoplankton (Fig. 1). These results suggest that the relative toxicity of different As species to freshwater phytoplankton decreased in the following order: As^V^ ≥ As^III^ ≥ DMAA^V^ ≥ AB. This also suggests that the growth inhibition of phytoplankton by As species depends on the phytoplankton strain, which is consistent with the results of other studies^11,26–28^. Some strains of freshwater phytoplankton are more resistant to As^V^ and As^III^ toxicity, while others are more susceptible. This might be due to them having differential abilities to detoxify and/or biotransform As^V^ and As^III^ into less toxic organoarsenic compounds^2,26^.

Phytoplankton reduce As^V^ to As^III^, which is then followed by the oxidative methylation of this As species to form intermediate trivalent methylAs species (MMAA^III^ and DMAA^III^) and pentavalent methylAs species (MMAA^V^ and DMAA^V^) in them^18^. A study by Hasegawa *et al*.^14^ reported that the freshwater phytoplankton *C*. *aciculare* converted approximately 80% of As^V^ into the less toxic DMAA^V^, with trace concentrations of trivalent methylAs species (MMAA^III^ and DMAA^III^) also formed. The biotransformation of pentavalent As^V^ by freshwater phytoplankton has been discussed in detail elsewhere^6^.

Based on the toxic effects (*i*.*e*. growth inhibition) of iAs species on them at high concentrations (≥10^4^ nmol L^−1^), the freshwater phytoplankton tested herein can be grouped into the following three distinct groups: (i) *B*. *braunii*, which is highly resistant to iAs and organoarsenic (orgAs) species; (ii) *S*. *actus* and *P*. *duplex*, which are highly resistant to As^III^ and orgAs species, as well as *S*. *paradoxum*, which is highly resistant to As^V^ and orgAs species; and (iii) *A. minutissium* and *C. aciculare* which are highly susceptible to iAs species (As^V^ and As^III^).

### Biotransformation of iAs species at different growth phases

#### Biotransformation of As^V^ into As^III^

**Figure 2:**
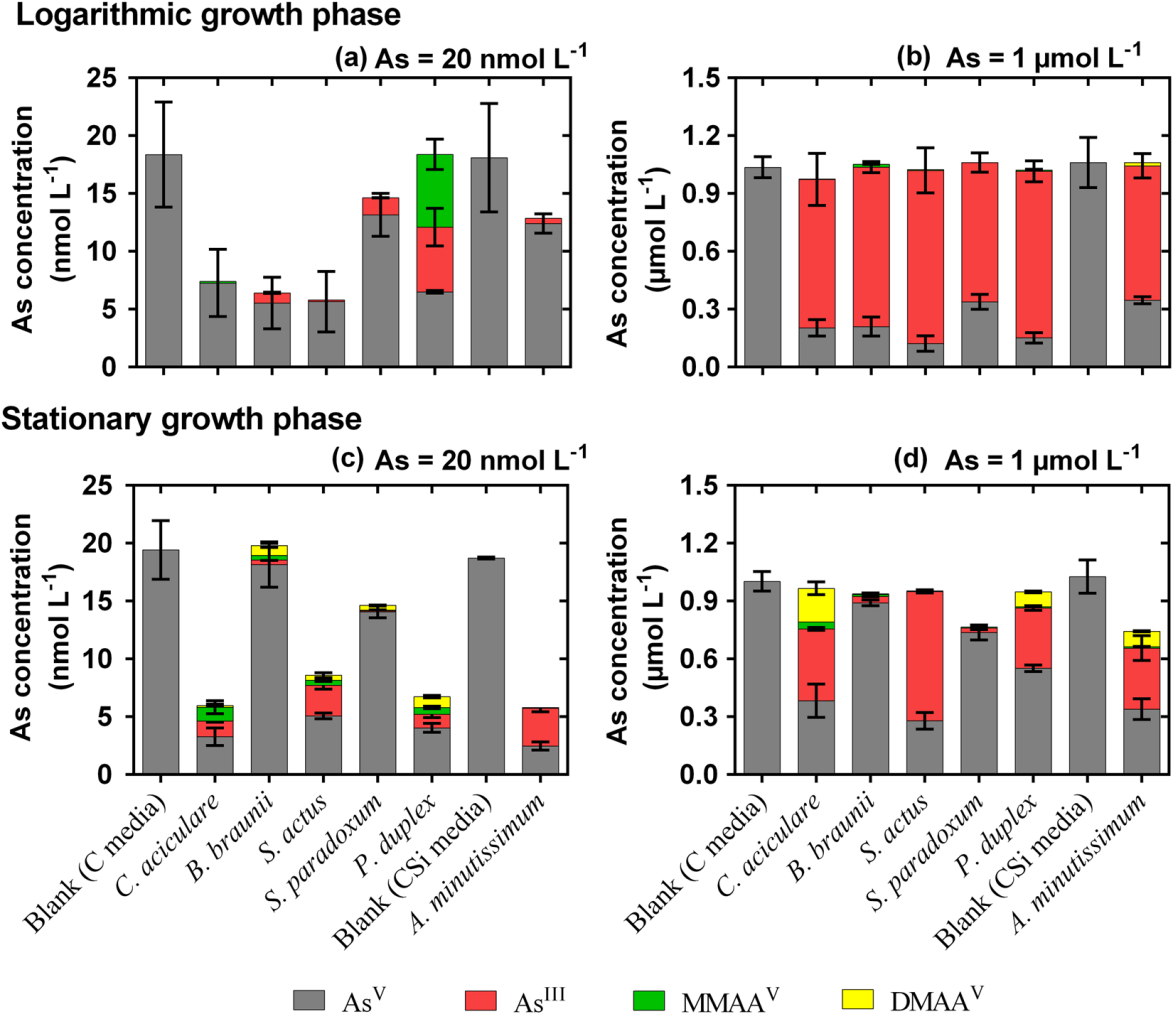
Arsenic speciation changes in the culture medium of freshwater phytoplankton at the logarithmic (**a**,**b**) and stationary (**c**,**d**) growth phases. Initially, the phytoplankton were grown in CSi culture medium with 20 nmol L^−1^ (**a**,**c**) and 1 μmol L^−1^ (**b**,**d**) of As^V^. Mean ± SD As concentrations are shown (n = 3).

The changes in As speciation in the phytoplankton culture media at the logarithmic and stationary growth phases at high and low As concentrations are shown in Fig. 2. These results reflect the bioaccumulation and biotransformation of As species by the phytoplankton inside their cells and their subsequent excretion into the environment. In general, in the low-[As]_0_ treatment (20 nmol L^−1^), As^V^ was the predominant species in the growth medium, followed by As^III^, at both the logarithmic and stationary growth phases (Fig. 2a,c). However, in the high-[As]_0_ treatment (1.0 µmol L^−1^), As^III^ was the predominant species in the medium at the logarithmic growth phase (Fig. 2b), while As^V^ was predominant at the stationary growth phase of all microalgae except *S*. *actus* (Fig. 2d).

**Figure 3:**
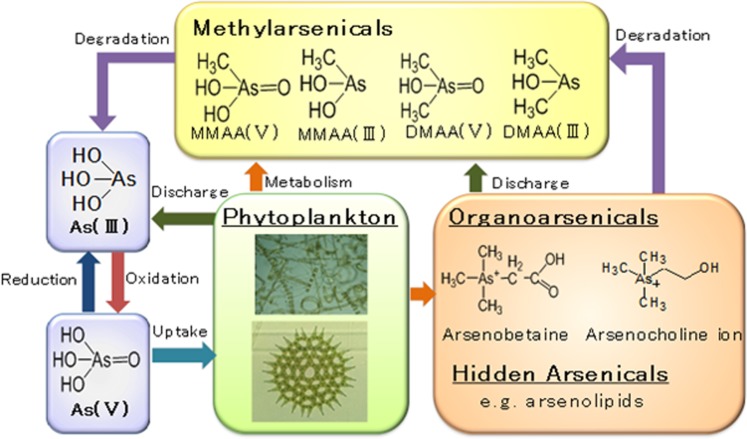
Biotransformation of As species by phytoplankton in aquatic systems.

Phytoplankton plays significant roles in the biotransformation and biogeochemistry of As in aquatic systems, as illustrated in Fig. 3. Because of the physicochemical similarities between arsenate and phosphate, phytoplankton actively uptake As^V^ through the PO_4_^3−^ uptake system^2,14,29^. In the present study, phytoplankton were exposed to NaH_2_AsO_4_·7H_2_O, and the concentration ratio of arsenate to phosphate in the growth medium was kept high to encourage arsenate uptake by phytoplankton. Arsenate has toxic effect on phytoplankton (as discussed in section 2.1.) due to the binding of AsO_4_^3−^ to places inside the cells where PO_4_^3−^ binding is essential^17^. The phytoplankton biotransform As^V^ into As^III^ inside their cells^26,27,30^, possibly to reduce the toxicity of As^V^ to them^2^. The biotransformation process involves the two-electron reduction of the pentavalent As^V^ to the trivalent As^III^, which is mediated by glutathione^18^.

The biotransformation of As^V^ into As^III^ and its subsequent methylation to form methylarsenic compounds (methylAs; *e*.*g*., DMAA and MMAA) and more complex organoarsenic compounds (orgAs; *e*.*g*., AB) by phytoplankton are correlated with the growth rate of the phytoplankton, and to their phosphorus nutrient status^17^. The phytoplankton take up higher amounts of As^V^ than PO_4_^3−^, reduce As^V^ to As^III^, and then excrete As^III^ out of their cells as their growth rates decline and under phosphate-depleted conditions^2^. As^III^ is further biotransformed into methylAs and more complex orgAs species, which are then excreted out of the cells. The present study further showed that the biotransformation of As^V^ into As^III^ by freshwater phytoplankton was slow at the logarithmic growth phase in the low-[As]_0_ treatment. However, most of the As^III^ was excreted out of the cells of the phytoplankton at this growth phase in the high-[As]_0_ treatment.

**Figure 4:**
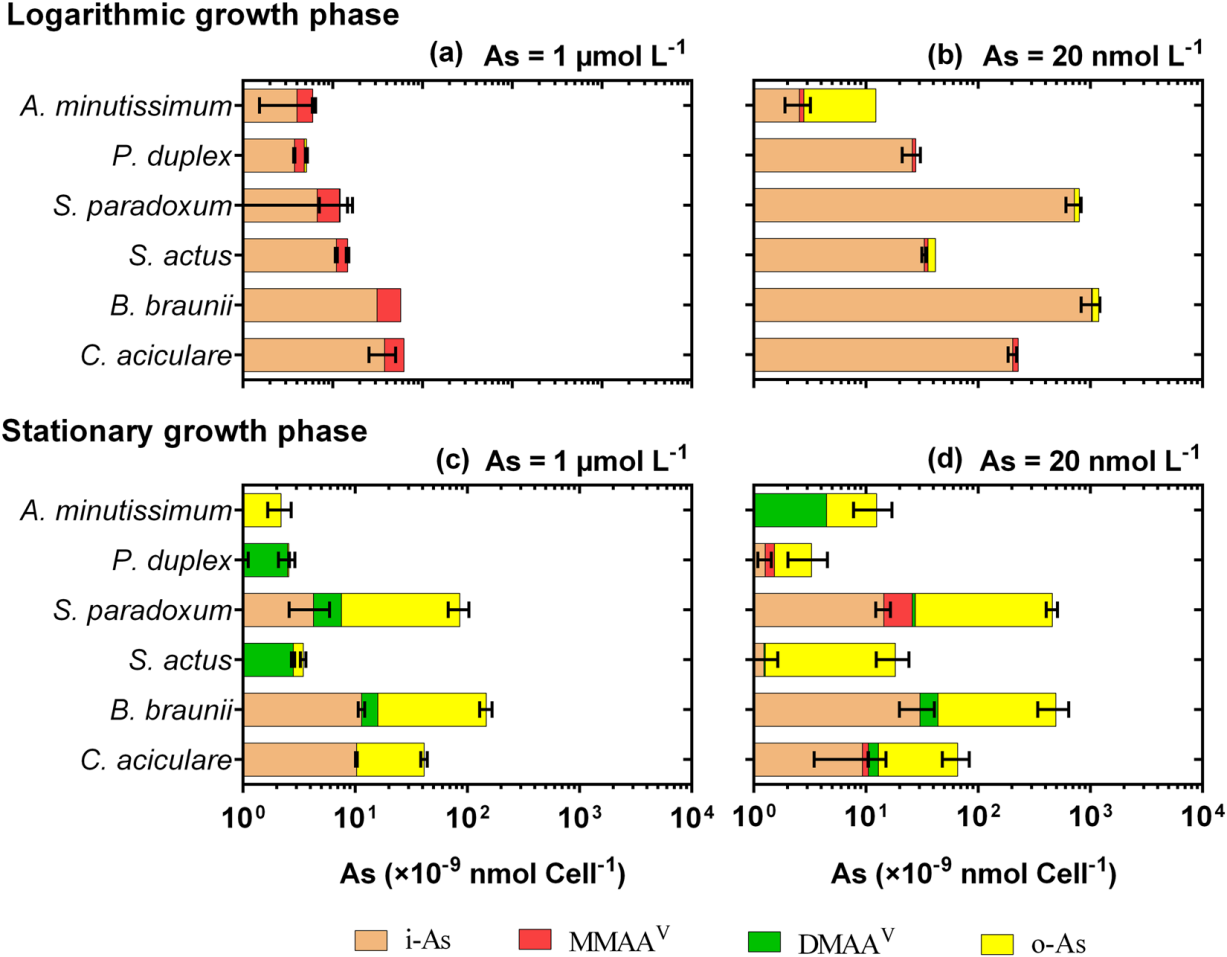
Arsenic speciation in freshwater phytoplankton cells at the logarithmic (**a**,**b**) and stationary (**c**,**d**) growth phases. Initially, the phytoplankton were grown in CSi culture medium with 1.0 µmol L^−1^ (**a**,**c**) or 20 nmol L^−1^ (**b**,**d**) of As^V^. Mean ± SD As concentrations are shown (n = 3).

In the high-[As]_0_ treatment, the dominant species of As in the growth medium at the logarithmic phase was As^III^ (Fig. 2b), whereas in the low-[As]_0_ treatment As^III^ was the elast prevalent in the medium at this stage (Fig. 2a). However, the relative abundances of As^V^ and As^III^ in the phytoplankton cells were similar in the high- and low-[As]_0_ treatments (Fig. 4a,b). These results demonstrated that the biotransformation of As^V^ into As^III^ inside the cells was not affected by the concentration of [As]_0_ in the surrounding medium. However, the excretion of As^III^ out of the cells was affected by the [As]_0_ in the surrounding medium. This might have been due to the fact that: (i) although As^III^ is more toxic than As^V^ is^31^, As^III^ is more easily excreted than As^V^ from the cells^2^; and/or (ii) the excretion of As^III^ out of the cells (efflux, which many researchers have agreed occurs to reduce the toxic effects of As^III^^2,8,17^) is negatively correlated to the As concentration in the surrounding medium due to equilibrium effects^27^. The present study also showed that, unlike in the high-[As]_0_ treatment, As^III^ excretion out of the phytoplankton cells in the low-[As]_0_ treatment happened at the logarithmic and stationary growth phases. However, the excretion rate of As^III^ was higher at the logarithmic phase than that at the stationary phase.

#### Biotransformation of iAs into methylAs and orgAs

Irrespective of the [As]_0_ treatment, iAs species (As^V^ + As^III^) were the predominant As species in the growth medium at the logarithmic growth phase (Fig. 2a,b). Intracellular As speciation results also showed that iAs species (As^V^ + As^III^) were the main As species inside the cells at the logarithmic growth phase, although a small amount of methylAs (mainly MMAA) was also found inside the cells at this growth phase (Fig. 4a,b). A significant proportion of the total As (t-As) inside the cells was represented by methylAs and orgAs species at the stationary growth phase (Fig. 4c,d), while the methylAs and orgAs concentrations in the growth medium were insignificant compared to the iAs concentrations therein (Fig. 2c,d). These results indicated that after the phytoplankton biotransformed iAs into methylAs and orgAs species inside their cells, these species were not excreted out of the cells by the organisms. This can be explained by the low toxicity and efflux rates of methylAs and orgAs species.

Inside the phytoplankton cells, As^V^ is reduced to As^III^, which is then followed by the oxidative methylation of intermediate trivalent methylAs species (MMAA^III^ and DMAA^III^) to form pentavalent methylAs species (MMAA^V^ and DMAA^V^)^18^. A number of freshwater phytoplankton have been reported to biomethylate iAs^19,22^. It is known that methylAs and orgAs species are less toxic than iAs species^31^. It is widely accepted that phytoplankton employ two main strategies to reduce the toxic effect of iAs: (i) the excretion of iAs (mainly As^III^) out of their cells; and (ii) the biotransformation of toxic iAs into less toxic methylAs and orgAs species^2^. As methylAs and orgAs species are less toxic to them, phytoplankton do not need to excrete them out of their cells, and therefore a significant amount of methylAs and orgAs were found inside the phytoplankton cells. This result also suggests that the methylation of As^V^ occurs more slowly than its reduction, and also differs among strains of freshwater phytoplankton.

### Diversity in As biotransformation by freshwater phytoplankton

**Figure 5:**
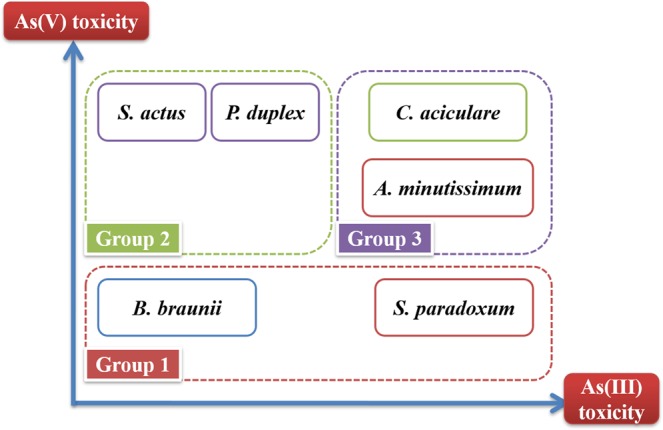
Diversity of As biotransformation by freshwater phytoplankton. All of the six-freshwater phytoplankton tested converted As^V^ into As^III^ at the logarithmic growth phase. However, the phytoplankton could be grouped into the following three groups based on their As biotransformation ability at the stationary growth phase. **Group 1:** *B*. *braunii* and *S*. *paradoxum* converted As^III^ into As^V^; **Group 2:** *S*. *actus* and *P*. *duplex* maintained As^V^ in their cells; and **Group 3:** *A*. *minutissium* and *C*. *aciculare* converted iAs into methylarsenic species.

An interesting pattern in the As biotransformation performed by the six freshwater phytoplankton strains studied in the present study was observed that potentially explains the diversity of As biotransformation by phytoplankton. Based on the biotransformation efficiency of different As species, the freshwater phytoplankton examined herein could be categorized into three groups: (i) phytoplankton that are efficient at biotransforming As^III^ into As^V^ (*e*.*g*., *B*. *braunii* and *S*. *paradoxum*); (ii) phytoplankton that cannot efficiently biotransform As, and thus rather maintain As^V^ inside their cells (*e*.*g*., *S*. *actus* and *P*. *duplex*); and (iii) phytoplankton that are efficient at biotransforming iAs into methylAs species (methylation) and complex orgAs species (*e*.*g*., *A*. *minutissium*, and *C*. *aciculare*) (Fig. 5; Groups A, B, and C, respectively).

Although there is no evidence to explain why different phytoplankton species have different As biotransformation efficiencies, this is likely related to the differential physiological activities of the phytoplankton, such as bioaccumulation, detoxification (transformation of toxic iAs species into less toxic methylAs and orgAs species), and excretion of As out of the cells. The fact that *B*. *braunii* and *S*. *paradoxum* (Group A phytoplankton; Fig. 5) convert As^III^ to As^V^ indicates that, once they are released into the oxic medium, then As^III^, MMAA, and DMAA are transformed back into the thermodynamically static As^V^. In terms of As^III^ biotransformation, the bio-oxidation process plays a vital role in freshwater algal cells, and the transportation of As^III^ takes place via aquaporins^32^. In the case of *S*. *actus* and *P*. *duplex* (Group B phytoplankton; Fig. 5), As^V^ was taken up simultaneously and accumulated inside the cell. The uptake of As^V^ by microalgae without transformation and their keeping it inside the cell likely occurs due to the detoxification strategies of these freshwater microalgae. The occurrence of this phenomenon in *S*. *actus* indicates that this species may be genetically incapable or energetically incompetent to take part in As biotransformation activities^33^. The subcellular distribution and chemical forms of heavy metals may play important roles in the metal tolerance and detoxification responses in plants^34^. The ability of *A*. *minutissium* and *C*. *aciculare* (Group C phytoplankton; Fig. 5) to transform As^V^ into As^III^ or methylated As forms confirm the occurrence of As biotransformation process in these microalgae. In general, bio-reduction occurs in association with the methylation process in response to As^V^ biotransformation. A study by Murray *et al*.^35^ on microalgae, including *D*. *salina* and *C*. *vulgaris*, provided evidence that this sort of biotransformation of As species could be performed by these microalgae, which was in agreement with the findings for phytoplankton Group C in the present study. Phytoplankton converted As^V^ to As^III^, and as the incubation period continued the As^III^ accumulated in the cells gradually began to undergo the methylation process, mainly to form DMAA, and was then likely excreted into the medium. The quantity of orgAs species present was related to the methylation/demethylation rate and release mechanisms of the microalgae. The presence (or absence) of MMAA in microalgal cell also confirmed the occurrence of the iAs methylation process in them. Therefore, biotransformation diversity among microalgae depends not only on the As speciation in the medium, but also on the As bioaccumulation and biotransformation methods used by each individual phytoplankton species.

The rates of As uptake by the different microalgae used in this study varied, suggesting that the consumption or sequestration of arsenic species depends on the structural and biochemical properties of microalgae, and differs among microalgae belonging to different species or classes. Uptake of As by cells depends on the valence of arsenic, as well as on several biotic and abiotic factors. Relevant biotic factors include the microalgal species and its uptake pathways, mode of detoxification, and whether it has had earlier exposure to As. On the other hand, abiotic factors include the As species, phosphate concentration, pH, and time of exposure. Several studies suggested that living biota (both aquatic and terrestrial) have various methods they can use to detoxify arsenic-like metals and metalloids. These may involve: As elimination from cells^36^, reduction of As^V^ to As^III^ ^37^, secretion of polychelatins (*e*.*g*., metal-binding proteins)^38^, and the subsequent methylation of As to form less toxic complexes^39^. Nevertheless, the detoxification processes carried out by microalgae, due to their impacts on the excretion of As^V^, As^III^, or organic As species^32^, have significant influences on their growth and As^V^ uptake and elimination.

This study showed that As biotransformation by and toxicity to freshwater phytoplankton was greatly influenced by the chemical species of As present, the type of phytoplankton species considered, and the phosphate concentration in the growth medium. Changes in experiment duration and conditions (such as exposure period and phosphate concentration) altered the behavior and As biotransformation processes carried out by the phytoplankton species. The growth rate of each species varied even when they were treated with the same experimental procedures, suggesting that each species has its own specific As uptake mechanism(s).

### A conceptual model of As metabolism in freshwater phytoplankton

**Figure 6:**
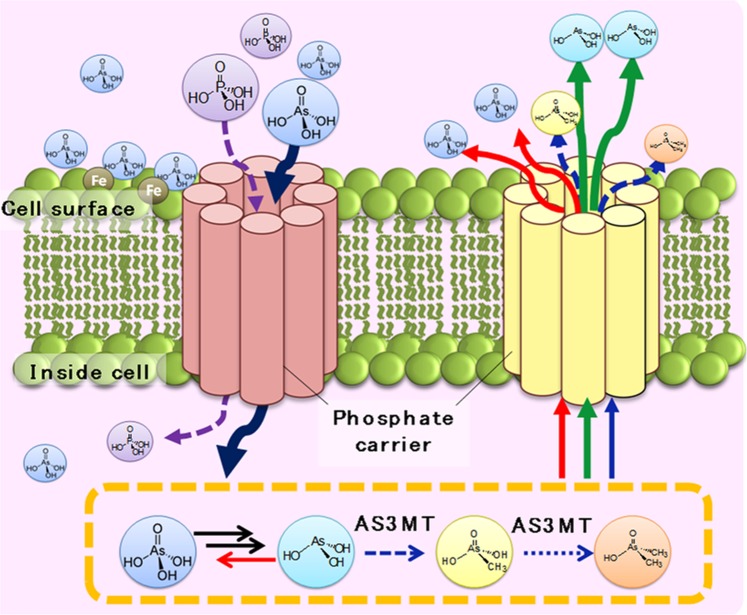
Conceptual model of As metabolism in freshwater phytoplankton. Arsenic resistance in this study depended on the kinds of freshwater phytoplankton considered. In the logarithmic growth phase, freshwater phytoplankton took arsenate into their cells and reduced it to arsenite. In the stationary phase, freshwater phytoplankton transformed the chemical forms of the arsenic in the surrounding environment, and then released it from their cells rapidly. The arsenic resistance of freshwater phytoplankton has an interactive relationship with the changing availability of different arsenic species in the hydrosphere.

A model of As biotransformation by freshwater phytoplankton based on the experimental results of this study is presented in Fig. 6. In this model, the As^V^ present in the medium is adsorbed on the cell surface, and is then taken into the cell via the PO_4_^3^^–^ transport system. This phenomenon happens due to the similar chemical properties of arsenate and phosphate. Arsenate can be actively taken up into the phytoplankton cells by the phosphate transporter pathway^40^ and competing with phosphate uptake^41^. This competitive uptake between As^V^ and phosphate in phytoplankton cells indicates the probable mode of action of the toxicity of As species. The As concentration in the cell proportionally increases with the As substrate concentration in the medium. This suggests that As uptake is influenced by metalloid availability in the environemnt^42–44^. As^V^ is reduced to As^III^ in the logarithmic growth phase and excreted into the growth medium via active transport. This reduction reaction may be carried out by thiols and/or dithiols, as As is likely to bind with the biochemical components of protein and non-protein thiols^39^. As^III^ is then methylated to form MMAA and DMAA, which then diffuse into the medium, suggesting that the chemical form of As species is changed depending on the As tolerance of the phytoplankton during the transition from the logarithmic growth phase to the stationary phase. Uptake of As^V^ causes the reduction of phosphate accumulation in the cell, likely due to As phytotoxicity or competitive uptake^45^. Inside the cell, PO_4_^3–^ groups are replaced by As^V^ in ATP, which forms an unstable ADP-As complex that interferes with other physiological systems, such as energy flow^46^.

Uptake of As^III^ into the cell occurs via aquaporin nodulin26-like intrinsic proteins (NIPs). Aquaporins are a kind of water channel protein that can transport extracellular water molecules into the cell. Several studies have suggested that As^III^ likely interferes with enzymatic reactions^47^ and the photosynthesis process^48^. Consequently, pigment, peptide, and lipid profiles can be altered in microalgal cells exposed to inorganic As species^49^. There are several steps involved in the metabolic transformation of iAs into methylated species, such as mono-, di-, and tri-methylated arsenic compunds^50^. Among these steps, some are associated with chemical reactions, whereas others are enzymatically catalyzed. It was previously suggested that arsenic methyltransferase (AS3MT) acts as a catalyst that leads to the transformation of iAs into methylated compounds. However, the methylated compounds yielded by this process are actually more reactive and toxic^51^.

The reduction of As^V^ to As^III^ occurs in the presence of several reductases that use glutaredoxin, glutathione, or thioredoxin as electron donors^32,52^. The methylation of As^III^ was reported to be slower than that of As^V^ to prevent As^III^ from building up inside the cell^17^. As^III^ is excreted less and its concentration gradually declines as a result of ongoing abiotic oxidation. MMAA and DMAA occur inside the cell due to the methylation of As^III^, and their excretion increases their concentrations in the culture medium. The production rates of DMAA and MMAA are comparatively slower than that of As^III^ at the logarithmic growth phase, likely due to the lower uptake of As^V^ at this phase^17^. The presence of phytochelatins (PCs) in microalgae leads to the binding of As to thio groups, for example glutathione (GSH), and thus PCs play important roles in As complexation and detoxification^53^. PC synthesis takes place at a rate adequate to bind the As in the cell at lower As concentrations^54^. The presence of intracellular GSH along with PCs permits the effective accumulation of As inside the cell^55^. The reduction of As^V^ to As^III^ with GSH as the electron donor in an aquatic medium promotes the formation of arsenotriglutathione complexes. These complexes readily donate As^III^ to targets including dithiols^56^. Hayakawa *et al*.^57^ reported that methylation mechanisms could occur via the formation of such complexes. The methyl group of S-adenosylmethionine (SAM) is transferred to As by AS3MT, and then the methylated compound undergoes hydrolysis and is oxidized, and the methylation process then proceeds sequentially. SAM is required for the As methylation reaction, in which it acts as a methyl donor, and if its availability changes this affects the patterns and extent of the As methylation process. The fact that the methylation mechanism of As involves AS3MT indicates that As methylation is an oxidative process. Chemically active oxygen was reported to initiate oxidative stress due to its involvement in the catalytic reaction of methyltransferases^58^. Free radicals are generated due to the reaction of dimethylarsine with molecular oxygen during the metabolic reduction of DMAA^III^. The mechanism of As uptake indicates that the pathway leading from iAs to methylated compounds includes several steps that generate several different intermediates and elements.

## Conclusion

This study reported the toxicity, biotransformation, and release of different As species in freshwater phytoplankton, which may help us to better understand the biogeochemistry of As in freshwater systems. Inorganic As^V^ was taken up by the phytoplankton, biotransformed inside their cells, and then released into the culture medium as As^III^ during the exponential growth phase at a high As^V^ to phosphate ratio. The growth inhibition effects of As^V^ and As^III^ were significantly higher than those of orgAs, DMAA^V^, and AB on the tested freshwater phytoplankton. The six freshwater phytoplankton strains examined could be categorized based on their As biotransformation patterns as follows: (i) those efficient in the biotransformation of As^III^ into As^V^; (ii) those not efficient in As biotransformation, which rather maintained As^V^ inside their cells; and (iii) those efficient in the biotransformation of iAs into methylAs species (methylation) and complex orgAs species. These results reflect the differential bioaccumulation and biotransformation of As species by these phytoplankton inside their cells and their excretion of these As species into the environment. Further work is needed to assess whether the toxicity of As species differs for other aquatic microorganisms such as zooplankton, to better understand the influence of As on freshwater ecosystem dynamics.

## Materials and Methods

### Reagents

Commercially available products were used as reagents without further purification in this study. Special-grade NaOH (NakaraiTesque, Kyoto, Japan) and HCl (Kanto Chemical, Japan) were used to adjust the pH of reagents and media. Special-grade disodium hydrogen formate heptahydrate (As^V^) and arsenic trioxide (As^III^) (Wako, Osaka, Japan), sodium cacodylate (DMAA^V^) (NacaraiTesque, Kyoto, Japan), and arsenobetaine (AB) (Tri Chemical, Yamanashi, Japan) were used for the modification of culture media, and 4-(2-hydroxyethyl)-1-piperazinyl ethanesulfonic acid (HEPES; NacalaiTesque, Kyoto, Japan) was used as a buffer reagent in culture media. KH_2_PO_4_ was purchased from Wako (Osaka, Japan) and ethylenediamine-N,N,N′,N′-tetraacetic acid (EDTA; >98.0%) was obtained from Dojindo, Japan. These reagents were diluted to the desired concentration in ultrapure water. Water was purified using ultrapure water production equipment (arium pro UV, Sartorius StedimBiotech GmbH) with a resistivity of 18.2 MΩ.

### Phytoplankton

Six strains of clonal axenic freshwater phytoplankton, namely *Achnanthidium minutissimum*, *Botryococcus braunii*, *Scenedesmus actus*, *Staurastrum paradoxum*, *Pediastrum duplex*, and *Closterium aciculare*, were used in the present study. The strains of *A. minutissimum*, *B. braunii*, *S. actus*, *S. paradoxum*, and *P. duplex* used were obtained from the National Institute for Environmental Studies, Japan. The tested strain of *C. aciculare* (isolated from Lake Biwa) was provided by Dr. Kanako Naito of Prefectural University of Hiroshima, Japan. The bioconcentration factors of the phytoplankton obtained under the same experimental conditions used in this study are provided in Table 1.

**Table 1:** Bioconcentration factor (BCF^a^) of freshwater phytoplankton used in this study.

Arsenic treatments (nmol L^−1^)	Culture period (days)	Chlorophyceae			Charophyceae		Bacilariophyceae
		*Botryococcus braunii*	*Scenedesmus actus*	*Pediastrum duplex*	*Closterium aciculare*	*Staurastrum paradoxum*	*Achnanthidium minutissimum*
Bioconcentration factors							
20	7	86.0	13.8	14.5	9.9	84.6	4.4
20	21	28.3	6.9	12.0	9.8	37.0	3.8
1000	7	12.8	1.5	1.0	0.3	8.9	0.3
1000	21	0.8	0.1	0.3	0.1	3.6	0.1

^a^$CF=\frac{{C}_{B}}{{C}_{W}}$, where, *C*_*B*_ is the intracellular arsenic concentration per 1.0 g of cell dry weight and *C*_*W*_ is the arsenic concentration in culture media.

*A*. *minutissimum* is distinguished from other monoraphid diatoms by its small size, linear-lanceolate shape, and radiate striae. Cells are solitary or form very short chains, and are often attached to the substrate by a stalk. This species’ distribution is biased towards alkaline waters, but it also appears in acidic waters^59^. It is widely adaptable to organic pollution, and dominates in rivers polluted by heavy metals^59^.

*B*. *braunii* is a pyramid-shaped planktonic green microalga that belongs to the family Botryococcaceae. This microalga inhabits freshwater, with cells with a diameter of 10–20 µm that form aggregated colonies. This species is able to produce hydrocarbons (particularly triterpenes), which comprise around 30–40% of its dry weight^60^. This organism synthesizes oil in its cells that is secreted extracellularly. The oil produced by *B*. *braunii* is expected to someday be used as an alternative fuel to gasoline.

*S*. *actus* is a green microalga belonging to the family Scenedesmaceae that has lanceolate cells. It always forms colonies, with colonies of 4 (or 2, 8, or 16) cells often connected in a line. It is widely distributed in freshwater environments, such as paddy fields, ponds, and swamps, as well as in soil. Its cells adhere to one another via the cell wall, and their positions do not change. In addition, the whole body may be wrapped in agar with extracellular polysaccharide secretions. The cells constituting the colonies have no flagella and are not motile.

*S*. *paradoxum* is a green microalga in the family Desmidiaceae with solitary floating cells. There is a constriction at the center of the cell, which divides each cell into two half-cells (desmids). The shape of the cell as seen from above (top-view) is a regular polygon. Four long arms extend from the central point. It is extremely common in various freshwater areas, such as lakes, ponds, paddy fields, rivers, and so on.

*P*. *duplex* is a species of freshwater green microalgae in the family Hydrodictyaceae. It forms colonies with specific numbers of cells (8 to 32 cells). The cell bodies are polygonal, granulated, and have horn-like projections. One cell has two protrusions, and there is a wide gap between the cells. The colonies are as large as single-celled algae, with a diameter that reaches tens to hundreds of microns, and the colonies have limited motility. This microalga is widely distributed in freshwater environments, such as paddy fields, ponds, and swamps. Most of these microalgae are free-floating, but there are also benthic forms.

*C*. *aciculare* is a crescent-shaped, unicellular, freshwater microalga. It can be found in almost all freshwater environments, from still-water ponds to running waters. There are 2 (rarely 4) chloroplasts in each cell, and they are divided in the center.

### Pre-culture and maintenance

CSi medium (Table 2) was used for the maintenance of the six studied freshwater phytoplankton strains. For the growth experiment, the phosphate concentrations of the CSi medium were adjusted to 1.0 μmol L^−1^ or 50 μmol L^−1^, and then media were modified by supplementation with arsenic solutions. The culture medium and apparatus (tips, bottles, vessels, and micropipettes) were sterilized separately at 121 °C for 30 min using an autoclave (MLS 3780, Sanyo Electric, Japan). They were then placed in a clean bench (NK Clean Bench, VSF-1300A, Nippon Medical Equipment Co., Japan) under UV irradiation for 20 min. Before using the phytoplankton in the trials, the cultures were maintained in identical media for 1–2 weeks in polycarbonate bottles (Nalge, USA) until they reached at least the exponential growth phase in a temperature- and light-controlled incubator (Koitotron3HN-35MLA, Koito Industries, Ltd. Japan). Experimental cultures were grown at 25 °C under a 12:12 h light/dark photoperiod, at a light intensity of 50 μE m^−2^ s^−1^ provided by cool white fluorescent lights. The axenic nature of the phytoplankton cultures was verified by performing the 4′,6-diamidino-2-phenylindole (DAPI) test and by the examination of cells under an epifluorescence microscope^61^.

**Table 2:** Chemical composition of CSi culture medium for freshwater phytoplankton used in this study.

Nutrients/Chemicals	Concentrations
Ca(NO_3_)_2_·4H_2_O	635 mmol L^−1^
KNO_3_	989 mmol L^−1^
KH_2_PO_4_	213 mmol L^−1^
MgSO_4_·7H_2_O	162 mmol L^−1^
MnCl_2_·4H_2_O	0.55 mmol L^−1^
ZnSO_4_·7H_2_O	0.23 mmol L^−1^
CoCl_2_·6H_2_O	0.05 mmol L^−1^
Na_2_MoO_4_·2H_2_O	2.73 mmol L^−1^
FeCl_3_	1.31 mmol L^−1^
Na_2_EDTA·2H_2_O	8.06 mmol L^−1^
Na_2_SiO_3_·9H_2_O	10 mg mL^−1^
Vitamin	1.0 × 10^−6^ mg mL^−1^
4-(2-hydroxyethyl)-1-piperazine ethane sulphonate	50 mg mL^−1^

### Growth experiments with various As concentrations

Phytoplankton cells acclimated to culture media at the exponential growth phase were incubated in 60-mL capacity polycarbonate vessels containing 60 mL of sterilized CSi culture medium (Table 2). The culture medium was then modified by changing the concentration of phosphate (added as KH_2_PO_4_) to encourage the uptake of arsenic species. Two different arsenic concentration treatments, high- arsenic ([As]_0_ = 1.0 μmol L^−1^) and low-arsenic ([As]_0_ = 20 nmol L^−1^), were used in the experiment, in which As was provided in the form of NaH_2_AsO_4_. Initially, the cell density in the culture medium was less than 4.6 × 10^3^ cells mL^−1^. After incubating the phytoplankton for three days, four species of arsenic (As^V^, As^III^, DMAA^V^, and AB) were added to the culture medium. The cultures were grown for 30 days. Phytoplankton growth was measured spectrophotometrically using a UV-VIS (ultraviolet-visible) spectrophotometer at 540 nm. The cell number was estimated with an established cell density-to-absorbance ratio. The number of cells was counted directly under a microscope. The growth rate (%) was defined by the following equation:

$${\rm{Growth}}\,{\rm{rate}}[ \% ]=({{\rm{OD}}}_{Sample}/{{\rm{OD}}}_{{\rm{Control}}})\times 100$$

where OD_*Sample*_ is the optical density of phytoplankton at 540 nm in arsenic-containing media and OD_*Control*_ is the optical density of phytoplankton in arsenic-free media after seven days of cultivation.

### Sample processing

On a pre-specified day, samples were collected and filtered through 0.45-μm membrane filters under low vacuum pressure (<30 mm Hg). The filtrate sample was then stored in a cool and dark place after adding 0.50 mL of 1.0 mol L^−1^ HCl to them. After that, 20 mL of 3.0 mol L^−1^ HCl was added to the sample, and the mixture was heated on a hot plate at 100 °C for 3 h to extract the arsenic both inside and outside of the freshwater phytoplankton cells. Thereafter, the mixture volume was adjusted to 40 mL with ultrapure water, and it was also adjusted to the same concentration as that of 1.3 mol L^−1^ HCl (pH = 0).

### Analysis of arsenic speciation in microalgal samples

**Figure 7:**
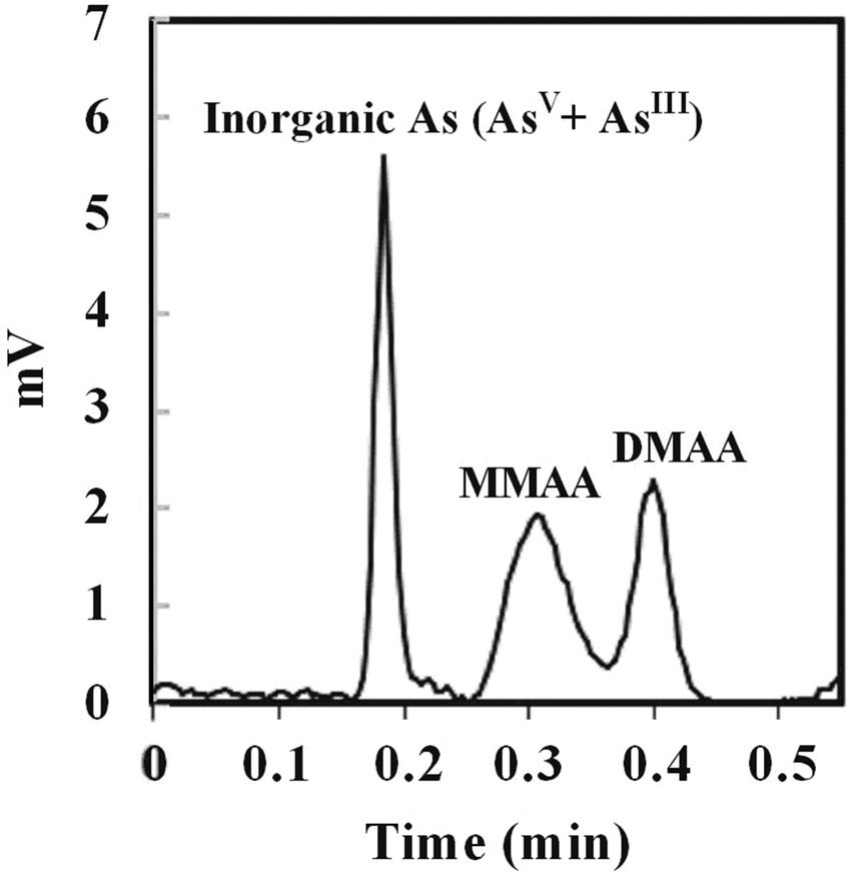
Chromatogram showing occurrence and separation of peaks representing each arsenic species for 20 nmol L^−1^ concentration sample.

A hydride generation technique was used for the determination of arsenic species in culture media according to Hasegawa *et al*.^62^. The technique was a combination of a flame atomic absorption spectrophotometer (AAS, 170-50 A, Hitachi, Japan) and hydride generation device followed by cold trapping^8,63^. Concentrations of iAs (As^V^ + As^III^), MMAA, and DMAA were analyzed by adding 5.0 mL of 0.20 mol L^−1^ EDTA·4Na and 5.0 mol L^−1^ HCl to 40 mL of the sample solution^3^. For As^III^, 5.0 mL of 0.20 mol L^−1^ EDTA·4Na and 0.5 mol L^−1^ KH_2_PO_4_ was added to 40 mL of the sample solution. The arsenic species detected were recorded in the form of a chromatogram using a data processing device (Chromato-PRO, Runtime Instruments, Tokyo, Japan), and their concentrations were determined based on the heights of the peaks obtained. A representative chromatogram of the occurrence and separation of peaks for each of the arsenic species is given in Fig. 7. The limit of detections (LODs) for the iAs (As^V^ + As^III^), MMAA, and DMAA were 0.3, 0.8 and 0.7 nmol L^−1^, respectively. The precisions as relative standard deviation (RSD, n = 5) for 20 nmol L^−1^ of iAs (As^V^ + As^III^), MMAA, and DMAA were 2.2, 1.2 and 1.4%, respectively. The accuracy of the analysis was checked using the certified standard reference material 1573a (tomato leaf from NIST, USA)^64^. The recovery of As concentration was 95.0% of the certified value.

### Determination of the total arsenic concentrations in cell samples

The total concentrations of arsenic in the freshwater microalgae were determined by inductively coupled plasma mass spectrometry (ICP-MS, SPQ 9000, SEIKO, Japan). Filter paper containing microalgal cells was digested by a microwave digestion system (Multiwave 3000, Anton Paar) using the Sea 1-h method. After that, digested liquors were co-washed with 15 mL of ultrapure water and transferred to heat-resistant DigiTUBEs (DigiPREP Jr, SCP SCIENCE). Tubes were then heated on hot plates at 100 °C for 6–7 h. After evaporation, 2 mL of deionized water was added to the samples, and they were then subjected to ICP-MS for the quantification of the total arsenic content in their cells. The operational conditions of the ICP-MS were the following: a high-frequency output of 1.2 kW; plasma gas flow rate of 16 L min^−1^; auxiliary gas flow rate of 1.0 L min^−1^; nebulizer gas flow rate of 1.0 L min^−1^; and sample replacement time of 10 s.

The mean values in different treatments were compared using Duncan’s multiple range test using the statistical program SPSS 22.0 for Windows (SPSS Inc., USA).
